# Environmental Enrichment Effect on Oxidative Stress in Hypertensive Rats

**DOI:** 10.5935/abc.20190221

**Published:** 2019-11

**Authors:** Marcelo Diarcadia Mariano Cezar, Aline Regina Ruiz Lima, Luana Urbano Pagan, Ricardo Luiz Damatto

**Affiliations:** 1 Faculdade de Ciências Sociais e Agrárias de Itapeva (FAIT), Itapeva, SP - Brazil; 2 Universidade Estadual Paulista (UNESP), Botucatu, SP - Brazil

**Keywords:** Rats, Hypertension, Stress, Oxidative, Cardiovascular Diseases/mortality, Renovascular Hypertension, Environmental Change

Arterial hypertension (AH) is frequently associated with metabolic disturbs, target-organs functional and/or structural alterations. In Brazil, it contributes, directly or indirectly, to 50% of cardiovascular disease death.^1^ AH experimental models have showed biochemical and cardiovascular alterations.^2-4^

Among the most common experimental models of AH in rats are genetic hypertension developed by Okamoto and Aoki^5^ with the spontaneous hypertension model, Dahl salt-susceptible, a result of a defect in renal excrete sodium, neurogenic hypertension, defined as a permanent increase in blood pressure resulting from a fundamentally neural (central or peripheral) change. Renal hypertension may be renoprive, produced by severe reduction in renal function, renovascular hypertension that is due to partial obstruction of blood flow to the kidneys, or in some cases both.^6^

Animal laboratory researches show that environmental enrichment stimulates species natural behavior, besides prevent stress signs, suffering and diseases, such as hypertension.^7^

Environmental enrichment, characterized by exposition to different stimulus, has the purpose to potentialize social interactions and development sensorial and motor stimulation.^7^ The enriched environment can induce beneficial effects similar to those promote by physical exercise on brain and behavior, both in humans and animals.^8^

Several studies have observed the environmental enrichment role on alterations due to AH.^9,10^ Pressoric overload is followed by redox imbalance, which is characterized by reactive oxygen species production and antioxidant capacity unbalance.^11^

The titled study “Environmental Enrichment Promotes Antioxidant Effect in the Ventrolateral Medulla and Kidney of Renovascular Hypertensive Rats”, published in this edition, aimed to evaluate the environmental enrichment on oxidative stress in ventrolateral bulb, heart and kidneys of renovascular-hypertension rats.^10^

Souza et al.^10^ study indicates similar mean arterial pressure (MAP) values between normotensive and hypertensive rats exposed to enriched environments. The authors highlight that it is the first study to demonstrate these results in hypertensive animals at these conditions.

Animals which stayed in enriched environment showed increased antioxidant enzymes activity, superoxide dismutase and catalase on the ventrolateral bulb, as well as TBARS (Thiobarbituric acid reactive substances) reduction. On kidneys, it was observed an increased superoxide dismutase activity. However, on the right kidney, it was found increased carbonyl protein concentration and lower TBARS concentration in hypertensive animals exposed to enriched environment. On the left kidney, hypertensive animals showed catalase enzyme activity reduction regardless of environment. Corroborating study data, oxidative profile alterations are found in young and old rats exposed to environmental enriched associated with antioxidant enzymes adaptations and oxidative damage reduction.^12^

Lacchini et al.^12^ assigned oxidative stress improvement to voluntary physical activity performed by animals exposed to enriched environment. In this study, enriched environment was not able to promote not promote cardiac left ventricle antioxidant enzymes and oxidative damage biomarkers alterations. Thus, it is likely that enriched environment including running wheels foment the physical activity required to antioxidant effects could be observed in heart.^7^

Wherefore, authors noted that enriched environment promotes antioxidant effects on ventrolateral bulb and kidneys, and it is a potential factor to reduce MAP and oxidative damage in renovascular hypertensive rats. Enriched environments studies that enable voluntary motor activity are needed for greater comprehension of the relationship between environmental enrichment and oxidative stress.
